# Computational and Transcriptome Analyses Revealed Preferential Induction of Chemotaxis and Lipid Synthesis by SARS-CoV-2

**DOI:** 10.3390/biology9090260

**Published:** 2020-09-01

**Authors:** Hibah Shaath, Nehad M. Alajez

**Affiliations:** 1 College of Health & Life Sciences, Hamad Bin Khalifa University (HBKU), Qatar Foundation (QF), PO Box 34110 Doha, Qatar; hshaath@hbku.edu.qa; 2 Cancer Research Center, Qatar Biomedical Research Institute (QBRI), Hamad Bin Khalifa University (HBKU), Qatar Foundation (QF), PO Box 34110 Doha, Qatar

**Keywords:** COVID-19, SARS-CoV-2, SARS-CoV, SARS-MERS, influenza A, influenza B, rhinovirus, Bioinformatics, gene expressions, pathway analysis, IFN response, immune response, fatty acid synthesis

## Abstract

The continuous and rapid emergence of new viral strains calls for a better understanding of the fundamental changes occurring within the host cell upon viral infection. In this study, we analyzed RNA-seq transcriptome data from Calu-3 human lung epithelial cells infected with severe acute respiratory syndrome coronavirus 2 (SARS-CoV-2) compared to five other viruses namely, severe acute respiratory syndrome coronavirus (SARS-CoV), Middle East Respiratory Syndrome (SARS-MERS), influenzavirus A (FLUA), influenzavirus B (FLUB), and rhinovirus (RHINO) compared to mock-infected cells and characterized their coding and noncoding RNA transcriptional portraits. The induction of interferon, inflammatory, and immune response was a hallmark of SARS-CoV-2 infection. Comprehensive bioinformatics revealed the activation of immune response and defense response to the virus as a common feature of viral infection. Interestingly however, the degree of functional categories and signaling pathways activation varied among different viruses. Ingenuity pathways analysis highlighted altered conical and casual pathways related to TNF, IL1A, and TLR7, which are seen more predominantly during SARS-CoV-2 infection. Nonetheless, the activation of chemotaxis and lipid synthesis was prominent in SARS-CoV-2-infected cells. Despite the commonality among all viruses, our data revealed the hyperactivation of chemotaxis and immune cell trafficking as well as the enhanced fatty acid synthesis as plausible mechanisms that could explain the inflammatory cytokine storms associated with severe cases of COVID-19 and the rapid spread of the virus, respectively.

## 1. Introduction

Recent events have highlighted the importance of studying viruses and their evolution over time in adapting to new hosts. Our understanding of the etiology of different viral infections has never been more imperative than it is today, in conjunction with building our knowledge on how different viruses establish uniqueness, leading to varied host ranges, severities, and longevities of infection. Additionally, identifying similarities between different viruses can be just as important in worldwide efforts to understand the disease and to develop vaccines efficiently and promptly in times of worldwide pandemics such as today.

A number of viruses have been implicated in human disease, manifesting in seasonal bouts, some reoccurring annually. Among the most common are the coronaviruses, including severe acute respiratory syndrome coronavirus (SARS-CoV), Middle East respiratory syndrome coronavirus (MERS-CoV), and the new coronavirus, severe acute respiratory syndrome coronavirus 2 (SARS-CoV-2); the influenza viruses, influenza A virus (FLUA) and influenza B virus (FLUB); and the rhinovirus (RHINO). Post-mortem examinations attribute respiratory viruses such as those mentioned to up to half of all sudden deaths in children [1], with seasonal pandemics claiming more lives each year. It is likely that sudden death is caused by a disproportionate inflammatory response to viral infection [2], leading to lung injury, viral sepsis, and respiratory distress [3]. This stresses the crucial need for more educated forecasts on potential upcoming viral trends, for better preparation and subsequent disease control worldwide.

Coronavirus, deriving from the *Coronaviridae* family, have positive-sense, single-strand RNA genomes encoding at least four structural proteins. The membrane (M) protein gives the virus its spherical virion structure. The envelope (E) protein is suggested to play a major role in the assembly and release of the virus coupled with its capacity for ion channel activity, facilitating pathogenicity [4,5]. The nucleocapsid (N) protein is highly phosphorylated for RNA binding. Importantly, it also binds to nsp3, a component of the replicase complex, which is important for encapsulating the genome into viral particles [6]. The spike (S) proteins have proven to be crucial for host cell entry. Homotetramers of this protein make the spike-like protrusions on the exterior of the virus for its initial attachment to receptors of the host cell, leading to infection. Extensive research on host receptors has been conducted, identifying human angiotensin converting enzyme 2 (hACE2) as a receptor for human viral entry for SARS-CoV and SARS-CoV-2 [7,8]. In the case of MERS-CoV, antibodies directed against human dipeptidyl peptidase 4 (hDPP4) inhibited the infection of primary human bronchial epithelial cells [9]. Rhinoviruses remain the primary cause of mild respiratory tract infections (the common cold) [10]. Upon infection, typical symptoms ensue such as inflammation and the release of cytokines such as IL-1, IL-6, IL-8, and TNF-α [11]. Similar to coronaviruses, rhinoviruses also have positive-sense, single-strand RNA genomes that, unlike the coronaviruses, are arranged within capsids (VP1, VP2, VP3, and VP4), rather than enveloped; therefore, they cannot undergo membrane fusion in the same way [12,13]. Depending on the human rhinovirus subtype, multiple mechanisms of viral host entry have been suggested. Receptors such as low-density lipoprotein receptor (LDLR) and intercellular adhesion molecule-1 (ICAM-1), which are expressed on nasal epithelial cells, have been proposed as mediators of receptor-mediated endocytosis in the major rhinovirus subtypes [14,15]. Some rhinovirus un-coating and the release of viral RNA into the host cytosol have been attributed to low endosomal pH [16].

The influenza virus is a rapidly evolving virus of four classes, influenzavirus A (FLUA), influenzavirus B (FLUB), influenzavirus C (FLUC) and influenzavirus D (FLUD); three of which are known to affect humans. More commonly referred to as ‘the flu’, FLUA alone is the primary cause of flu pandemics, constantly evolving between 18 distinct subtypes of hemagglutinin (HA) and 11 distinct subtypes of neuraminidase (NA) glycoproteins, accounting for the variations in severity and transmissibility [17]. For example, the most common type flu of 2009 was H1N1 (swine flu), which is also responsible for the Spanish flu of 1918, one of the deadliest pandemics in history [18]. FLUB has a much lower mutation rate than FLUA; it is dominant in only one in every seven flu seasons [19,20]. All influenza viruses are similar in structure and composition [21]. Similar to the coronaviruses, they have envelop structures that home the viral genome, which in the case of influenza is comprised of several negative-sense RNA single strands [21]. The surface glycoproteins (NA and HA) facilitate target host cell entry and viral genome release into the host cytoplasm [22]. After the binding of HA antigens to the surface of epithelial cells in the nose, throat, and lungs, its cleavage initiates endocytosis for the initiation of viral replication [23].

We recently performed an in-depth molecular analysis utilizing transcriptome data from primary normal human bronchial epithelial cells (NHBE) during SARS-CoV-2 infection. Our study revealed an induction of inflammatory response and interferon response as the hallmark of SARS-CoV-2 infection of NHBE cells and COVID-19-derived tissue [24]. However, whether the observed molecular changes in host cells were unique to SARS-CoV-2 infection are not addressed thus far. In the current study, we compared six datasets of Calu-3 human lung epithelial cells infected with different viruses: SARS-CoV-2 [25], SARS-CoV, SARS-MERS [26], FLUA, FLUB, and RHINO [27]. Previous studies showed the suitability of the Calu-3 cell model due to its susceptibility to infection by respiratory viruses, including influenza virus A, rhinovirus, and SARS-CoV. Calu-3 polarize into stable cultures more rapidly than NHBE cells, which can differentiate into multiple cell types and layers, while Calu-3 cells culture in monolayers more efficiently. When subjected to infection, the Calu-3 cell model did not appear to alter tight junction formation, even though infection was found to be persistent for at least 6 weeks, providing a more stable and malleable model for studying viral infection, such as SARS-Cov-2 [28]. Our computation analysis provides a side-by-side comparison of molecular changes in host cells in response to SARS-CoV-2 and the aforementioned respiratory viruses. While Calu-3 cells exhibited common altered functional categories and signaling pathways in response to various viruses, there were remarkable differences in the magnitude of the response. Our study provides insight into the pathogenesis and host response to novel coronavirus (SARS-CoV-2) compared to other common viruses, and it highlights chemotaxis and lipid synthesis as the main affected functional categories.

## 2. Materials and Methods

### 2.1. Culture Conditions and Viral Infection

A detailed methodology for SARS-CoV-2-infected cells can be found in Blanco-Melo et al., 2020 [25]. Briefly, Calu-3 were maintained at 37 C and 5% CO2 in Dulbecco’s Modified Eagle Medium (DMEM, GIBCO) supplemented with 10% Fetal Bovine Serum (FBS, Corning). SARS-CoV-2, isolate USA-WA1/2020 (NR-52281), was propagated in Vero E6 cells in DMEM supplemented with 2% FBS, 4.5 g/L D-glucose, 4 mM L-glutamine, 10 mM Non-Essential Amino Acids, 1 mM Sodium Pyruvate, and 10 mM HEPES. Total RNA from SARS-CoV-2 and mock-infected cells (24 h) was extracted using an RNeasy Mini Kit (Qiagen, Hilden, Germany), according to the manufacturer’s instructions. RNA-seq libraries of polyadenylated RNA were prepared using the TruSeq Stranded mRNA Library Prep Kit (Illumina, San Diego, CA, USA) according to the manufacturer’s instructions. Sequencing libraries were sequenced on an Illumina NextSeq 500 platform. For both SARS-CoV and MERS-CoV, detailed methodology can be found in Yeung et al., 2016 [26]. In brief, the EMC/2012 strain of MERS-CoV and the HKU39849 strain of SARS-CoV were propagated in VeroE6 cells, American Type Culture Collection (ATCC) in Dulbecco’s Modified Eagle Medium (DMEM) supplemented with 10% fetal calf serum (FCS) and 100 units mL^−1^ penicillin plus 100 µg mL^−1^ streptomycin (1% PS). Calu-3 cells were cultured in ATCC-formulated Eagle’s Minimum Essential Medium (EMEM) supplemented with 10% FCS and 1% PS. For RNA isolation, total RNA was isolated from mock or virally infected cells (24 h) using Trizol (Life Technologies Inc., Waltham, MA, USA). Sequencing libraries were prepared using a TruSeq Stranded mRNA Sample Prep Kit and sequenced on a HiSeq 2000 (Illumina, San Diego, CA, USA). Influenza A and B, and the rhinovirus preparation methodologies are described in detail in Dissanayake et al., 2020 [27]. Viruses were isolated from patients in Hong Kong with Influenza A (H1N1) virus A/HK/415742/2009 and influenza B virus B/HK/411989/2011, and a patient with pneumonia from which rhinovirus species A type 1A was isolated. Both influenzas were propagated in Madin Darby canine kidney (MDCK) cells at 37 °C, while rhinovirus was propagated in rhabdomyosarcoma RD cells at 33 °C. Viruses were resuspended in 1 mL of minimum essential medium (MEM) and Dulbecco’s Modified Eagle Medium (DMEM) for influenza virus and Rhinovirus, respectively. Calu-3 cells were infected with FLUA, FLUB, and rhinovirus in DMEM-F12 medium. RNA isolation was performed using a QIAamp R Viral RNA Mini Kit (Qiagen, Hilden, Germany). Total RNA was extracted from replicates for virally and mock-infected (control) at 24 h post-infection using RNeasy (Qiagen, Hilden, Germany). One microgram of total RNA was used as the starting material for cDNA libraries, which were prepared using a KAPA Stranded mRNA-Seq Kit (Roche, Mannheim, Germany) followed by paired-end Illumina sequencing.

### 2.2. Dataset and Bioinformatics

Raw RNA sequencing data were retrieved from the sequence read archive (SRA) database under accession nos. (SARS-CoV-2: SRP253951 [25]; SARS-CoV and SARS-MERS: SRP056612 [26]; FLUA, FLUB, and RHINO: SRP251473) [27]. Data were retrieved using the SRA toolkit version 2.9.2 as previously described [29]. FASTQ files were subsequently aligned to the hg38 reference genome using a built-in RNA-seq analysis module in CLC genomics workbench 20.0 with default settings, as we described before [30]. Expression data (TPM (Transcripts Per Million) mapped reads) were subsequently subjected to differential gene analysis using 2.0-fold change and a < 0.05 false discovery rate (FDR) adjusted *p*-value cut-off. Transcripts with raw expression values < 1.0 TPM were excluded from the analysis. Hierarchical clustering was performed using cosine for columns and cosine for rows, and marker finder prediction was used as described before using AltAnalyze v.2.1.3 [31,32,33].

### 2.3. Gene Set Enrichment and Modeling of Gene Interactions Networks

To minimize the effects of variations in basal gene expression in Calu-3 cells across the three different datasets, each of the virally infected Calu-3 cells was compared to their control from the same study. Differentially expressed genes from each study (log2 FC) were subsequently subjected to IPA analysis. Differentially expressed genes were imported into the Ingenuity Pathways Analysis (IPA) software (Ingenuity Systems; www.ingenuity.com) and were subjected to functional annotations and regulatory network analysis using upstream regulator analysis (URA) to analyze upstream molecules, which are connected to genes in the dataset via a set of either direct or indirect relationships based on changes in expression. Mechanistic networks (MN) analysis was employed by IPA to generate signaling cascades that connect upstream regulators to help visualize how they connect to explain the observed changes in gene expression. Downstream effector analysis (DEA) identifies the biological processes (disease) and functions, which are casually affected by the deregulation of genes in datasets and predicts their activation state (Z score). IPA uses precise algorithms to predict functional regulatory networks from gene expression data and provides a significance score for each network according to the fit of the network to the set of focus genes in the database. The *p*-value is the negative log of P, and it represents the possibility that focus genes in the network are found together by chance [30]. The comparison analysis feature in IPA was employed to enable the visualization of differentially affected cellular processed and pathways in Calu-3 cells following infection with the aforementioned viruses. Affected functional categories and pathways exhibiting ≥ 2.0 Z score (absolute) and *p* < 0.05 in at least one of the conditions were considered significant and were retained.

### 2.4. Statistical Analyses

Statistical analyses and graphing were performed using Microsoft excel 2016 and GraphPad Prism 8.0 software (GraphPad, San Diego, CA, USA). A two-tailed t-test was used for comparative groups. *p*-values ≤ 0.05 (two-tailed t-test) were considered significant. For IPA analyses, a Z score (2.0 ≤ Z ≤ −2.0) was considered significant.

## 3. Results

### 3.1. Alterations in Host Gene Expression in Calu-3 Cells in Response to SARS-CoV-2

**Figure 1:**
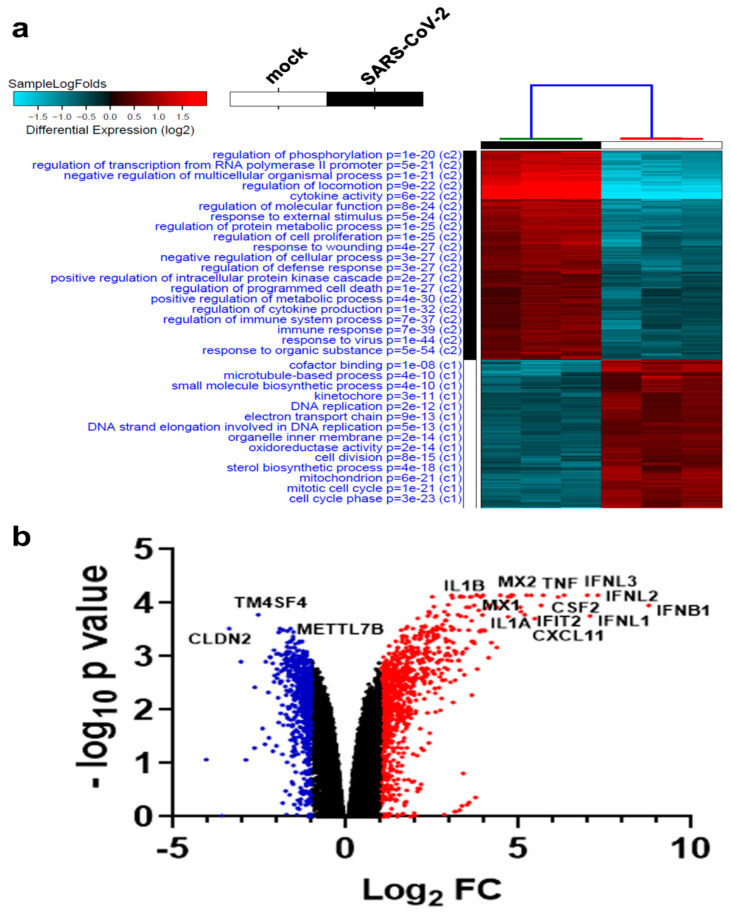
Clustering of severe acute respiratory syndrome coronavirus (SARS-CoV-2) and control Calu-3 cells based on RNA-seq analysis. (**a**) Hierarchical clustering of control and severe acute respiratory syndrome coronavirus 2 (SARS-CoV-2)-infected Calu-3 cells based on differentially expressed genes. Each column represents one replica, while each row represents one mRNA. Expression is depicted at the indicated color scales. (**b**) Volcano plot representation of significantly altered genes in Calu-3 SARS-CoV-2 vs. mock-infected cells. Red and blue colors indicate the genes with significantly increased or decreased expression, respectively.

To identify molecular changes in host genes due to viral infection, RNA-seq expression data retrieved from Calu-3 cells infected with SARS-CoV-2 were mapped to the h38 human genome assembly and subjected to hierarchical clustering. Eight hundred and thirty-three genes were upregulated, and 593 were downregulated post-infection with SARS-CoV-2 (Supplementary Table S1). Figure 1a highlights the most affected functional categories in SARS-CoV-2 infected Calu-3 cells. Enriched gene ontologies (GO) appear in red, whereas those suppressed are seen in blue. In agreement with our recently published data in NHBE cells during SARS-CoV-2 infection, most of the activated functional categories in response to SARS-CoV-2 were those involved in response to virus and immune response, while functional categories related to cell cycle and cell division were severely suppressed [24]. Several of the upregulated genes were indicative of interferon response (i.e., IFNB1, IFNL2, IFNL1, IFNL3, IFIT2, IFIT3, and others), immune response (i.e., CXCL8, CXCL10, CXCL11, CCL5, and others) or inflammatory response (i.e., TNF, IL-6, IL1A, IL1B, and others). A volcano plot (scatterplot) that shows statistical significance (-log10 p value; *Y*-axis) versus magnitude of change (log2 fold change; *X*-axis) is depicted in Figure 1b, with selected genes being displayed. The red colors (right) and blue colors (left) represent genes with significantly upregulated or downregulated expression in Calu-3 SARS-CoV-2 versus mock-infected cells, respectively (Figure 1b).

### 3.2. Alterations in Host Gene Expression in Calu-3 Cells in Response to SARS-CoV, SARS-MERS, FLUA, FLUB, and RHINO Infection

**Figure 2:**
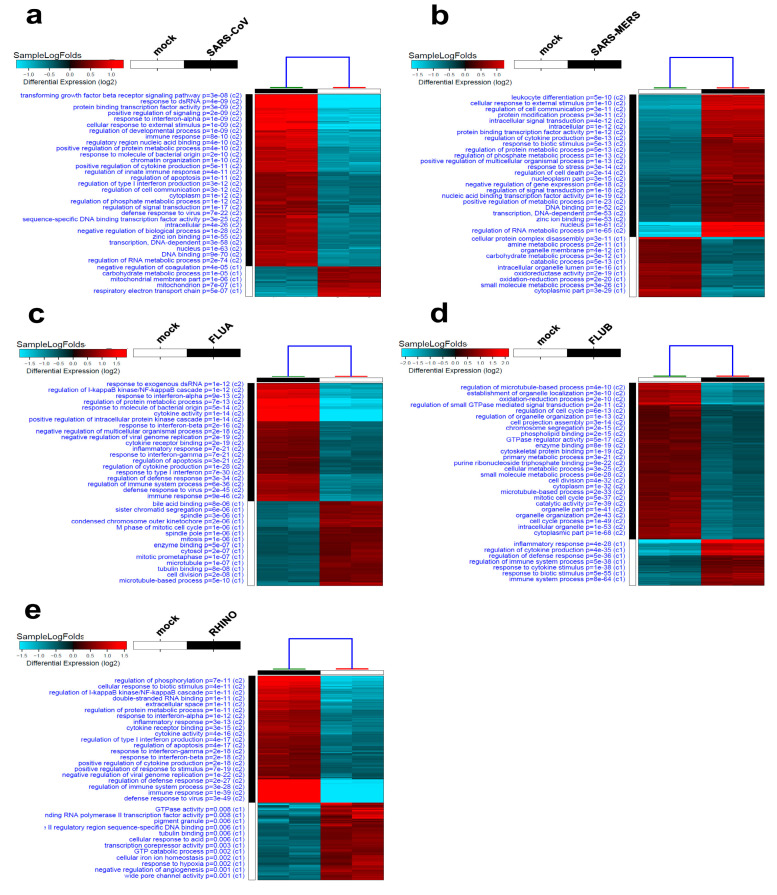
Hierarchical clustering and gene ontology (GO) enrichment of Calu-3 cells infected with different viruses. SARS-CoV (**a**), SARS-MERS (**b**), influenzavirus A (FLUA) (**c**), influenzavirus B (FLUB) (**d**), and rhinovirus (RHINO) (**e**). Affected gene ontology (GO) category in each experimental group is indicated on the left side. Each column represents one replica and each row represents a single gene. The expression level for each gene is depicted according to the color scale. MERS: Middle East respiratory syndrome.

RNA-Seq data for Calu-3 cells infected with SARS-CoV, SARS-MERS, FLUA, FLUB, and RHINO viruses were subsequently retrieved and were mapped to the h38 human genome assembly and were subjected to hierarchical clustering, as shown in Figure 2. GO analysis of SARS-CoV-infected Calu-3 cells indicated an activation of processes related to the regulation of the RNA metabolic process, DNA binding and defense response to virus, and the suppression of mitochondrial categories (Figure 2a). Overall, we observed a larger number of genes to be upregulated (1426 upregulated versus 225 downregulated; Supplementary Table S2) compared to changes observed in response to SARS-CoV-2 infection. Hierarchical clustering and GO analysis of the third coronavirus, SARS-MERS, highlights the activation of regulation of RNA metabolic process and DNA binding, similar to what was seen in SARS-CoV-infected cells (Figure 2b). The list of differentially expressed genes in response to SARS-MERS infection is shown in Supplementary Table S3. Aberrant gene expression in cellular hosts upon FLUA infection showed 661 upregulated and 473 downregulated genes (Supplementary Table S4). Hierarchical clustering and GO analysis showed the activation of cellular processes related to immune response and defense response to virus, as well as the suppression of microtubule and cell division processes (Figure 2c). Calu-3 cells infected with FLUB exhibited 1308 upregulated and 4435 downregulated genes (Supplementary Table S5), hence exhibiting the most alteration in gene expression in Calu-3 cells. Most of the activated cellular processes were those involved in the immune system process, in response to cytokine stimulus and inflammatory response, while most of the suppressed cellular processes were those involved in cell cycle regulation (Figure 2d). Lastly, characterizing the transcriptional landscape of RHINO-infected Calu-3 cells identified 382 upregulated genes versus 154 that were downregulated (Supplementary Table S6). Among the upregulated processes, defense response to virus and immune response were heightened, as well as multiple processes found in common with other viruses such as inflammatory response, cytokine activity, and general immune response. Figure 2e also highlights some of the suppressed processes in RHINO-infected cells, including GTPase activity, tubulin binding, and response to hypoxia. Taken together, our data revealed a considerable overlap in affected cellular processes upon infection with various viruses, mostly processes related to immune responses and cytokine activity, inclusive of interferons, and responsive to external stimuli.

### 3.3. Canonical Analysis Highlights Similarities and Differences in Pathways Activation in Response to Different Viruses

**Figure 3:**
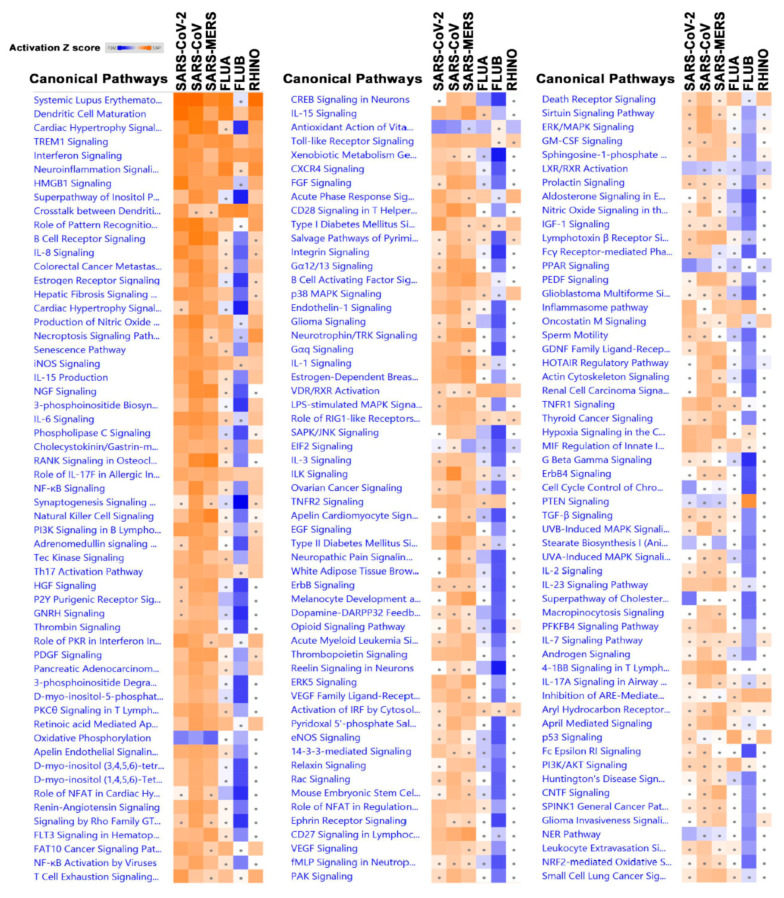
Significantly affected canonical pathways based on differentially expressed genes in the indicted treatment group (SARS-CoV-2, SARS-CoV, SARS-MERS, FLUA, FLUB, and RHINO) in Calu-3 cells. Differentially expressed genes from the six virally infected Calu-3 cells were subjected to canonical pathway analysis in ingenuity pathway analysis (IPA). Activation Z score is depicted according to the color scale (2.0 ≤ Z score ≤ −2.0). Red indicated activation, while blue indicated suppression. Squares with a filled circle denote canonical with an absolute activation Z score < 2.0.

Differentially expressed genes in Calu-3 cells infected with each virus were subsequently subjected to canonical pathway analysis using the ingenuity pathway analysis (IPA) bioinformatics tool. A unique pattern in canonical pathways for FLUB is instantly clear (Figure 3), where the majority of conical pathways are suppressed (in blue), according to the color scale (2.0 ≤ Z score ≤ −2.0). However, the activation of interferon signaling and crosstalk between dendritic cells and natural killer cells as well as other pathways can be seen in FLUB-infected Calu-3 cells. The full list of conical pathway analysis is listed in Supplementary Table S7. In general, the other five viruses (SARS-CoV-2, SARS-CoV, SARS-MERS, FLUA, and RHINO) seem to have similar canonical pathway patterns, whether activated or suppressed.

Of the most activated pathways with higher Z scores, dendritic cell maturation and interferon signaling was found in all six viruses. Other commonly significantly enriched pathways include Triggering Receptor Expressed On Myeloid Cells 1 (TREM1) signaling, nitric oxide synthase (iNos) signaling, and a heightened role of pathways involved in IL-17F in allergic inflammatory airway diseases (Figure 3). Some commonly suppressed conical pathways in the all six groups include those related to oxidative phosphorylation, the antioxidant action of vitamin C, peroxisome proliferator-activated receptor (PPAR) signaling, and Liver X Receptor-Retinoid X Receptor (LXR/RXR) activation. Evidently, some pathways are predicted to be activated by some viruses and not others; for example, viruses of the coronavirus family exhibited unity in some predicted pathway functions. All three coronaviruses exhibited enrichment in most canonical pathways, but they were uniform in pathways that were suppressed such as Phosphatase and tensin homolog (PTEN) signaling, which was significantly activated in FLUB infection. Interestingly, however, a higher degree of enrichment was observed in SARS-CoV-2 in infected cells in dendritic cell maturation, TREM1 signaling, High Mobility Group Box 1 (HMGB1) signaling, crosstalk between dendritic cells and natural killer cells, the role of pattern recognition receptors in the recognition of bacteria and viruses, IL-8 signaling, and several others (Figure 3 and Supplementary Table S7).

### 3.4. Significantly Affected Casual and Upstream Regulator Networks in Calu-3 Cells Infected with the Indicated Viruses Based on Transcriptome and IPA Analyses

**Figure 4:**
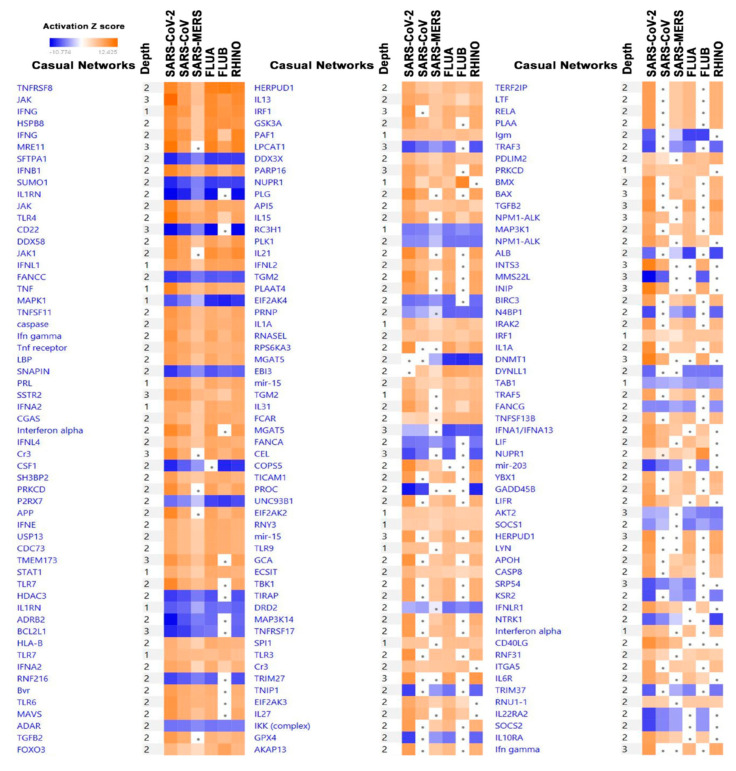
Significantly affected casual networks based on differentially expressed genes in the indicted treatment group (SARS-CoV-2, SARS-CoV, SARS-MERS, FLUA, FLUB, and RHINO) in Calu-3 cells. Differentially expressed genes from the six virally infected Calu-3 cells were subjected to casual network analysis in IPA. Activation Z score is depicted according to the color scale (2.0 ≤ Z score ≤ −2.0). Red indicates activation, while blue indicates suppression. Squares with a filled circle denote canonical with an activation Z score < 2.0.

Causal network analysis (CNA) identifies causal relationships associated with our data to include regulators that are not directly connected to targets in the data. Analysis on aberrantly expressed genes in response to viral infection predicts which casual networks are affected by these changes in expression. Among these networks, less disparity was observed compared to the conical network analysis, as all pathways seemed to be either uniformly activated or suppressed across the six groups of infected cells (Figure 4). The 10 most activated networks across all six infections include.

TNFRSF8, JAK, IFNG, HSPBB, MRE11, IFNB1, TLR4, DDX58, JAK1, and IFNL1, whereas the top 10 most repressed include SFTPA1, SUMO1, IL1RN, CD22, FANCC, MAPK1, SNAPIN, CSF1, P2RX7 and HDAC3. Some of the networks exhibited a higher degree of activation is SARS-CoV-2 compared to other viruses. For instance, JAK, MRE11, IFNB1, TLR4, TNF, MAKPK1, INFG, IFNA, TLR7, and several others were more activated in SARS-CoV-2 infected Calu-3 cells compared to other viruses (Figure 4 and Supplementary Table S8).

**Figure 5:**
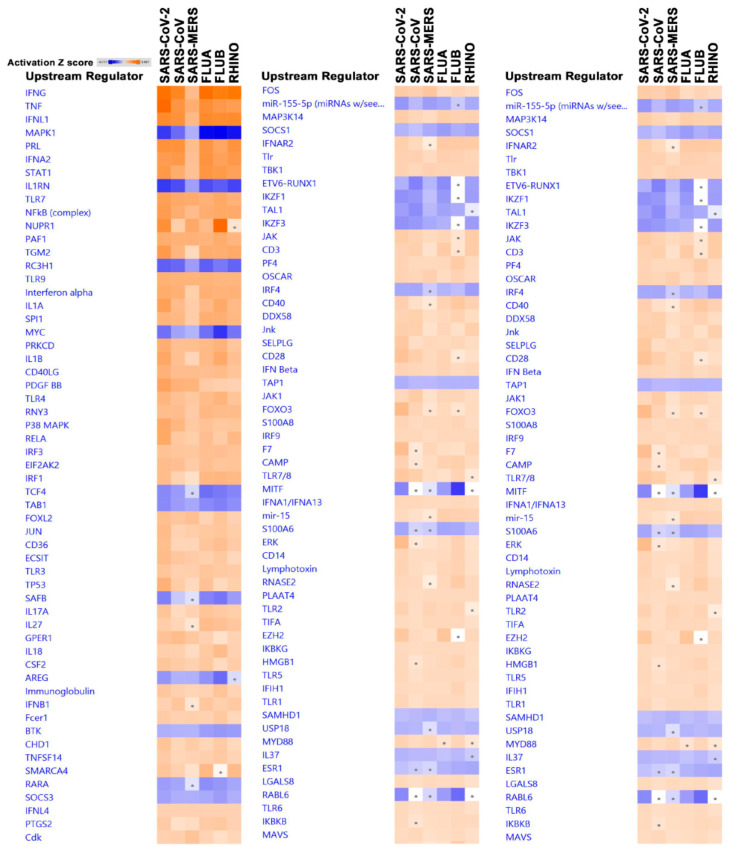
Significantly affected upstream regulator analysis based on differentially expressed genes in the indicted treatment group (SARS-CoV-2, SARS-CoV, SARS-MERS, FLUA, FLUB, and RHINO) in Calu-3 cells. Differentially expressed genes from the six virally infected Calu-3 cells were subjected to upstream regulator analysis in IPA. Activation Z score is depicted according to the color scale (2.0 ≤ Z score ≤ −2.0). Red indicates activation, while blue indicates suppression. Squares with a filled circle denote canonical with an activation Z score < 2.0.

Upstream regulator analysis enables us to predict the cascade of upstream transcriptional regulators that could have resulted in the observed gene expression changes in our data. Several of the observed upstream regulators are known to be associated with immunity and inflammation (Figure 5 and Supplementary Table S9).

IFNG, TNG, IL1A, IL1B, RELA, P38 MAPK, ERK, NFkb complex and several Toll-like receptors were significantly activated in our dataset. Suppressed upstream regulators include MAPK1, IL1RN, RC3H1 and MYC, which are common to all six datasets from the six Calu-3 cells infected with SARS-CoV-2, SARS-CoV, SARS-MERS, FLUA, FLUB, and RHINO versus mock-infected cells. However, and as observed in the canonical and casual network analysis, a higher activation of TNF, TGM2, IL1A, IL1B, RELA, and other pathways was observed in SARS-CoV-2 infected Calu-3 cells.

### 3.5. Alterations in Disease and Function Categories Based on Transcriptome and IPA Analysis of Infected Calu-3 Cells

**Figure 6:**
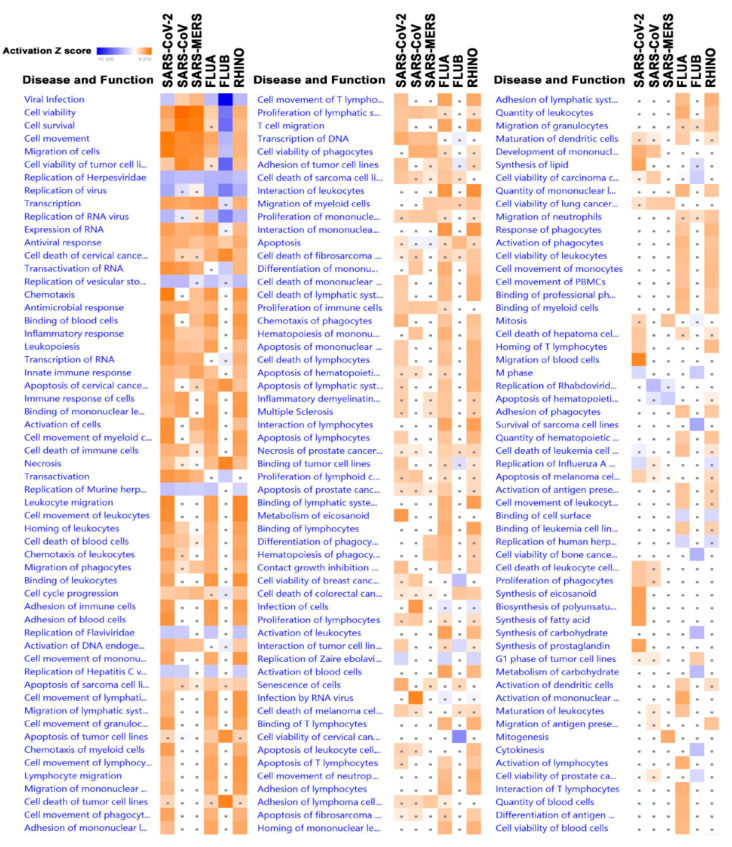
Significantly affected disease and function categories based on differentially expressed genes in the indicted treatment group (SARS-CoV-2, SARS-CoV, SARS-MERS, FLUA, FLUB, and RHINO) in Calu-3 cells. Differentially expressed genes from the six virally infected Calu-3 cells were subjected to disease and function analysis in IPA. Activation Z score is depicted according to the color scale (2.0 ≤ Z score ≤ −2.0). Red indicates activation, while blue indicates suppression. Squares with a filled circle denote canonical with an activation Z score < 2.0.

**Figure 7:**
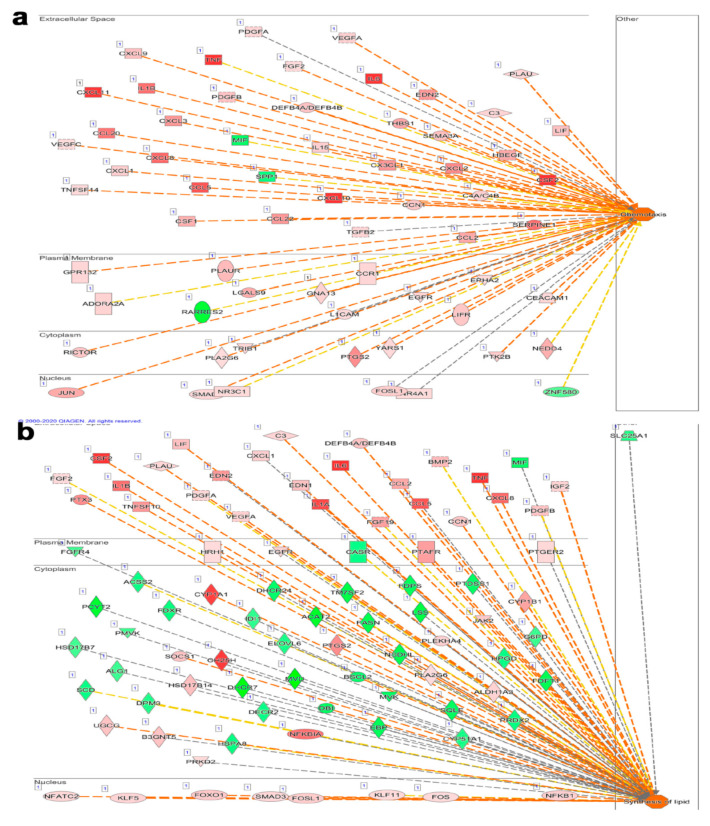
Activation of chemotaxis and synthesis of lipids functional categories in SARS-CoV-2-infected Calu-3 cells. Illustration of enriched chemotaxis (**a**) and fatty acid synthesis (**b**) functional categories in SARS-CoV-2-infected Calu-3 cells. Genes within the network, their interaction state, as well as their subcellular localizing based on IPA are indicated.

Our computational analyses revealed several functional categories to be affected as a result of the viral infection of Calu-3 cells. Figure 6 shows a plethora of categories, both activated and suppressed, with varied activation Z scores. Overall, the majority of functional categories were similarly affected by the six types of viruses. However, our data identified several functional categories that were differentially affected by individual viruses. For instance, several functional categories were severely suppressed by the FLUB virus, which were otherwise activated by the other five viruses (i.e., cell viability, survival, movement, and transactivation of RNA; Figure 6). Functional categories that are universally upregulated in response to all six viral infections include antiviral response, cellular homeostasis, and the migration of phagocytes. Those functions are suppressed by all six viral infections, with variable degrees, and include replication of the RNA virus. A remarkable observation from the disease and function analysis highlighted the significant activation of several disease and function categories in SARS-CoV-2-infected cells compared to all other virus-infected Calu-3 cells. Precisely, a higher activation Z score was observed for cell movement, migration of cells, chemotaxis, cell movement of myeloid cells, leukocyte migration, cell movement of granulocytes, cell movement of phagocytes, as we all as many other categories, all implying a robust immune response against SARS-CoV-2. The chemotaxis disease function category is illustrated in Figure 7a and Supplementary Tables S10 and S11.

Additionally, we observed the prominent activation of cellular functions related to the synthesis of lipid, synthesis of eicosanoid, synthesis of fatty acid, and synthesis of prostaglandin (Figure 7b and Supplementary Table S11). Collectively, our findings imply enhanced lipid synthesis and a possible connection to viral replication and budding.

## 4. Discussion

Our current study integrated multiple datasets and bioinformatical approaches to provide a comprehensive and thorough comparison of the cellular host response to SARS-CoV-2, SARS-CoV, SARS-MERS, FLUA, FLUB, and RHINO, which are a number of major viruses affecting world health today. Previous work by Yeung et al., reported renal infection and an induction of apoptosis in MERS-CoV-infected renal cells, which would be concordant with our findings from the current analysis of Calu-3 cells [26]. Canonical pathway analyses in the IPA of SARS-CoV-2 infected cells highlight a higher degree of enrichment in pathways related to dendritic cell (DC) maturation. DCs are important antigen-presenting cells in the initiation of an immune response, enabling the priming and activation of T cells to fight foreign infection [34]. A successful virus is likely to take steps in preserving their host and maintaining their replication reservoir; thus, most viruses will allow for a host immune response after the release of new virions into the host microenvironment [35]. A particularly strong host immune response through the enriched maturation of DCs can also lead to autoimmune complications such as those seen in COVID-19. Among the top upregulated genes in the dendritic cells category in SARS-CoV-2 Calu-3 cells from the current study were IFNB1, CSF2, TNF, IL-6, IL1A, IL1B, NFKBIA, ICAM1, LTB, IL12A, STAT1, CREB5, STAT2, and TNFRSF11B. Therefore, and while those genes are known to be involved in the maturation and function of dendritic cells, apparently, those genes could also be expressed by epithelial cells in response to viral infection. It is possible that the secretion of several of those chemokines (i.e., TNF, IL-6, IL1A, IL1B, and IL12A) by virally infected cells can orchestrate dendritic cell recruitment and function. For instance, Chomarat and colleagues reported that IL-6 can divert the differentiation of monocytes from dendritic cells to macrophages [36]. Interestingly, single-cell analysis of bronchial lavage from COVID-19 patients revealed the presence of monocyte-derived FCN1+ macrophages in severe COVID-19 patients, suggesting a possible role of IL-6 secreted by SARS-CoV-2-infected lung cells in promoting FCN1+ macrophages differentiation from monocytes [37]. In another study, TNF-α was shown to play a crucial role in the maturation of DC after adenovirus challenge and the activation of adaptive immunity in response to viral infection [38]. Therefore, it is plausible that cytokine secretion by SARS-CoV-2 infected epithelial cells can shape the immune response against SARS-CoV-2 and possibly other viruses, in part through the activation of macrophages and DCs. Aside from the DCs category, IL-8 signaling was also activated. Among its many functions, IL-8 plays a role in the recruitment of eosinophils, neutrophils, and naïve T cells to the sites of infection, acting as a chemoattractant [39]. The resulting elevated levels of cytokines have shown adverse effects in the context of SARS-CoV-2 infection. Cytokine storm syndrome occurs when large numbers of cytokine-releasing cells are recruited, eliciting a systemic inflammatory response that when dysregulated, can cause hypotensive shock and multi-organ failure [40]. Increasing evidence has shown that cytokine storms may be fueling some COVID-19 deaths. In April 2020, the Pediatric Intensive Care Society in the United Kingdom observed children presenting multi-system inflammatory state requiring intensive care. Another report describes cases in the US, where patients with post-infectious cytokine release syndrome had significantly longer bouts of fever [41].

Our data elude to a strong interferon response upon SARS-CoV-2 viral infection, as we have described earlier in Vishnubalaji et al., employing the NHBE cell model [24]. Our data suggests that both cell models, derived from human lung epithelial cells, can be used interchangeably in research, with the added advantage of Calu-3 lung adenocarcinoma cells accessibility and rapid generation with large numbers. Several of the identified genes in the current study has also been validated by other groups. A study by Lau et al. on cell lysates of Calu-3 cells infected with MERS-CoV or SARS-CoV showed significantly upregulated levels of IL-6 in MERS-CoV compared to SARS-CoV, while TNF, IFNB, and CXCL10 were greatly induced upon SARS-CoV infection compared to MERS-CoV, which would be concordant with data presented in the current study [42]. The expression of several cytokines identified in the current study (i.e., IL1B, IL-6, TNF, CCL2, CXCL9, and CXCL10) were also seen in bronchoalveolar lavage (BAL) cells from COVID-19 patients, providing further support to our observation [37]. The further validation of key genes during the course of SARS-CoV-2 infection will provide us with a clearer picture into key cytokines involved in the cellular host response to SARS-CoV-2 infection.

Canonical and casual network analysis gave us further insights into the pro-inflammatory environment upon viral infection, highlighting several Toll-like receptors, interferons, NFkB subunits, and tumor necrosis factors (TNFs) to be particularly activated in SARS-CoV-2 infection, positively correlating with disease severity. Our data are in line with Blanco-melo et al., who reported an induction of interferon response in Calu-3 cells infected with SARS-CoV-2 [25]. Anti-TNF treatment has even been suggested for the treatment of COVID-19, attenuating disease progression and leading to milder outcomes by suppressing systemic auto-inflammatory responses [43,44]. Similarly, IFN-α2b treatment of COVID-19 patients as a single agent or in combination with arbidol significantly reduced the duration of SARS-CoV-2 in the upper respiratory tract and reduced the duration of IL-6 and C-Reactive Protein (CRP) inflammatory markers in the blood [45].

Significantly affected disease and function categories also imply an activated immune response against SARS-CoV-2. Heightened chemotaxis functionality is particularly important to highlight, as this confirms the likelihood of macrophages and granulocytes recruitment to sites of infection, leading to the activated interferon response eluded to by the conical and casual pathway analysis in the current study. In line with our findings, recent single-cell analysis has highlighted the presence of neutrophils and macrophages in BAL as the hallmark associated with severe COVID-19 cases [37]. A study by Rzepka et al. looked at the immune response to Rat coronavirus (RCoV) viral infection through the response of polymorphonuclear leukocytes (PMNs) in type I-like alveolar epithelial (AT1) cells. Interestingly, several CXC chemokines were found to be required for the chemotaxis of PMNs toward RCoV-AT1 but not mock-AT1 [46], in concordance to our data, which identified several CXC chemokines to be associated with chemotaxis related to SARS-CoV-2 infection. These include CXCL3, CXCL8, CXCL9, CXCL10, and CXCL11 in the extracellular space of Calu-3-infected cells (Figure 6a). Furthermore, the neutralization of both CXCL1 and CXCL3 in the RCoV study showed a reduction in PMN chemotaxis, but not in the complete blockage [46], indicating a possible redundancy in some chemokines. Other potential chemotaxis-promoting factors also need to be explored in order to understand the exact mechanisms of chemotaxis and how they contribute to processes affecting the progression of viral infection. Alveolar macrophages secreting pro-inflammatory cytokines signals for the further chemotactic recruitment of monocytes and neutrophils, resulting in dysregulated inflammation. This lack of control prompts further macrophage infiltration, which could develop into lung injury through host organ attack and viral sepsis, creating even more complications for the patient infected [47]. Bouhaddou et al. provided a global phosphorylation overview and reported the production of a diverse number of cytokines as well as p38 MAPK and CK2 activation upon SARS-Cov-2 infection of vero E6 cells and reported morphological changes in SARS-Cov-2-infected cells—more specifically, actin-rich filopodial protrusions that were significantly longer and branched compared to normal cells, which also possessed budding viral particles [48]. Concordantly, in the current study, we observed a strong activation of the P38/MAPK pathway in SARS-CoV-2 infected Calu-3 cells (activation Z score = 5.8, *p* = 2.5 × 10^−27^) as well as the activation of CK2 (CSNK2A1; activation Z score = 3.6, *p* = 6.9 × 10^−36^) employing transcriptome and computational analyses.

Disease and function category analysis also predicted the prominent activation of cellular functions related to the synthesis of lipids, eicosanoids, fatty acids, and prostaglandin in response to SARS-CoV-2 infection. As one would expect, lipid interactions have been shown to play major roles during virus entry and infection. Lipids make up the main components of membranes and energy storage vesicles in eukaryotes, making them the first line of defense when encountering a pathogen [49,50]. Targeting lipid signaling allows viruses to remodel host cells, making them the most optimal for their own benefit [51]. The surge in lipid biosynthesis also serves to provide membranes for ‘safe’ viral replication and assembly and subsequent exit through budding, enhancing viral spreading, as seen particularly in the case of SARS-CoV-2 infection [52]. In addition to this, some viruses can encase themselves in lipid structures to avoid the recognition of their viral proteins, evading immune system recognition and prolonging its survival [51]. In fact, Dengue virus (DENV) has been proven to even hijack the host fatty acid synthase to sites of its own viral replication to further synthesize fatty acids [53]. Further research into the nature of virus–host lipid interactions can provide us with important targets, whereby anti-viral therapeutics targeting lipid metabolism can be potentially utilized for the control of viral infectious disease.

Recent clinical trials have confirmed the relevance of our data, highlighting the use of interferons as promising therapies for intervention in COVID-19 patients. In one study, 20 patients received 44 µg of IFN-β-1a subcutaneously every other day up to 10 days, along with conventional therapy (Clinical trial registration number: IRCT20151227025726N12). Within this time, virology clearance had decreased in time and there has been significant recovery in all patients within 14 days, supporting the use of IFN-β-1a in combination with hydroxychloroquine and lopinavir/ritonavir in the management of COVID-19 [54]. An early administration of TNFα inhibitor therapy in patients with severe COVID-19 infections has been proposed in the reduction of the viral burden, reducing the need for advanced cardiorespiratory support and preventing early mortality. Infliximab and Infliximab-abda to be used in this study are TNFα inhibitors currently FDA-approved for the treatment of autoimmune disorders, including Crohn’s disease and rheumatoid arthritis (ClinicalTrials.gov Identifier: NCT04425538).

Duvelisib, used to treat chronic lymphocytic leukemia or small lymphocytic lymphoma, has been proposed for phase II trials by investigators hypothesizing that PI3K inhibition with duvelisib could potentially subdue aberrant hyperactivtation of the innate immune system, reducing pulmonary inflammation and limiting viral persistence (ClinicalTrials.gov Identifier: NCT04372602). Our data can provide further insight for the development of additional therapies targeting the use of interferons and blockers of inflammatory mediators in the treatment of COVID-19. Altered conical and casual pathways related to TNF, IL1A, and TLR7, seen more predominantly during SARS-CoV-2 infection in our study, are just some potential targets for further investigations to assess their potential utilization in future clinical trials. Although findings from the current study remains to be validated experimentally, our findings serve as the basis for future work in the understanding of host response to SARS-CoV-2 and other viruses.

## 5. Conclusions

In summary, we have established the aberrant transcriptional landscape of Calu-3 host cells in response to six different viruses (SARS-CoV-2, SARS-CoV, SARS-MERS, FLUA, FLUB, and RHINO). Although we observed a large degree of overlap between affected genes ontologies and functional categories, SARS-CoV-2 in particular exhibited an enhanced activation of chemotaxis and lipid synthesis functional categories. These functions have been previously implicated in the progression of viral infection, namely via the recruitment of cytokine secreting cells leading to inflammation-induced complications such as tissue injury. The viral manipulation of lipid synthesis pathways have also been implicated in the masking of viral proteins, facilitating optimal conditions for viral replication and secretion through budding. Our findings from the current study provides novel insights into SARS-CoV-2 infection for better management of the disease and the development of effective therapeutic interventions.

## Supplementary Materials

The following are available online at https://www.mdpi.com/2079-7737/9/9/260/s1, Table S1: Differentially expressed genes in Calu3 cells infected with SARS-CoV-2, 2.0 FC, *p* (corr) < 0.05, Table S2: Differentially expressed genes in Calu3 cells infected with SARS-CoV, 2.0 FC, *p* (corr) < 0.05., Table S3: Differentially expressed genes in Calu-3 cells infected with SARS-MERS, 2.0 FC, *p* (corr) < 0.05, Table S4: Differentially expressed genes in Calu-3 cells infected with FLUA, 2.0 FC, *p* (corr) < 0.05, Table S5: Differentially expressed genes in Calu-3 cells infected with FLUB, 2.0 FC, *p* (corr) < 0.05., Table S6: Differentially expressed genes in Calu-3 cells infected with RHINO, 2.0 FC, *p* (corr) < 0.05, Table S7: Canonical pathway comparison for differentially expressed genes in Calu-3 cells infected with the indicated virus based on IPA analysis, Table S8: Casual network pathway comparison for differentially expressed genes in Calu-3 cells infected with the indicated virus based on IPA analysis, Table S9: Upstream regulator analysis comparison for differentially expressed genes in Calu-3 cells infected with the indicated virus based on IPA analysis, Table S10: Disease and function comparison for differentially expressed genes in Calu-3 cells infected with the indicated virus based on IPA analysis, Table S11: Affected disease and function categories in SARS-CoV-2 infected Calu-3 cells.
